# Gut Microbiota May Not Be Fully Restored in Recovered COVID-19 Patients After 3-Month Recovery

**DOI:** 10.3389/fnut.2021.638825

**Published:** 2021-05-13

**Authors:** Yu Tian, Kai-yi Sun, Tian-qing Meng, Zhen Ye, Shi-meng Guo, Zhi-ming Li, Cheng-liang Xiong, Ying Yin, Hong-gang Li, Li-quan Zhou

**Affiliations:** ^1^Institute of Reproductive Health, Center for Reproductive Medicine, Tongji Medical College, Huazhong University of Science and Technology, Wuhan, China; ^2^School of Basic Medicine, Tongji Medical College, Huazhong University of Science and Technology, Wuhan, China

**Keywords:** SARS-CoV-2, recovered COVID-19 patient, gut microbiota, 16S sequence, short chain fatty acids

## Abstract

Coronavirus disease 2019 (COVID-19) has infected over 124 million people worldwide. In addition to the development of therapeutics and vaccines, the evaluation of the sequelae in recovered patients is also important. Recent studies have indicated that COVID-19 has the ability to infect intestinal tissues and to trigger alterations of the gut microbiota. However, whether these changes in gut microbiota persist into the recovery stage remains largely unknown. Here, we recruited seven healthy Chinese men and seven recovered COVID-19 male patients with an average of 3-months after discharge and analyzed their fecal samples by 16S rRNA sequencing analysis to identify the differences in gut microbiota. Our results suggested that the gut microbiota differed in male recovered patients compared with healthy controls, in which a significant difference in Chao index, Simpson index, and β-diversity was observed. And the relative abundance of several bacterial species differed clearly between two groups, characterized by enrichment of opportunistic pathogens and insufficiency of some anti-inflammatory bacteria in producing short chain fatty acids. The above findings provide preliminary clues supporting that the imbalanced gut microbiota may not be fully restored in recovered patients, highlighting the importance of continuous monitoring of gut health in people who have recovered from COVID-19.

## Introduction

The pandemic of COVID-19, caused by severe acute respiratory syndrome coronavirus 2 (SARS-CoV-2), has resulted in more than 124 million confirmed cases according to Johns Hopkins University Data Archive (1), and the impact of COVID-19 on global public health could last for years to come. Therefore, it is of utmost importance to identify any potential sequelae and long-term effects given the large population of recovered patients worldwide. Physiologically, the infecting of cells with SARS-CoV-2 is mediated by the receptor angiotensin converting enzyme 2 (ACE2) and cofactor transmembrane serine protease 2 (TMPRSS2) (2). In addition to lungs, intestinal tract tissues also express a high level of ACE2 and TMPRSS2 (3). Clinical evidence indicated that gastrointestinal symptoms are common in COVID-19 patients (4) and SARS-CoV-2 RNA was detected in the fecal samples and gastrointestinal tissues (3, 5). The direct infection of the human intestinal tract and the active replication of SARS-CoV-2 were also established in the model of intestinal organoids (6). These results together support the impairment of intestines caused by SARS-CoV-2.

Notably, recent studies have demonstrated that COVID-19 inpatients have significant alterations in gut microbiota, consisting of an increase of opportunistic pathogens and loss of commensal bacteria (7–9). Several bacteria have been identified as biomarkers to distinguish healthy people and COVID-19 patients, and an abundance of specific microbes was found to be associated with disease severity, indicating the role of gut microbiota in the progression of COVID-19 (8, 10). The perspective that gut microbiota functioned in regulating host immunity and balancing inflammation has been accepted widely. This immunologic modulatory function is mainly executed by metabolites of microbes, such as short-chain fatty acids (SCFAs) (11). Changes in microbiota may cause abnormal intestinal immunity, which may lead to susceptibility to some diseases and involvement in their pathogenesis (12). The alternation of gut microbiota was also confirmed in other respiratory virus infections, which was recognized as the possible cause of intestinal inflammation and immune injury in influenza patients (13). Because of the hundreds of millions of recovered patients, the potential long-standing effect of infection on gut microbiota has attracted worldwide attention. However, current studies have centered mostly on the inpatients with COVID-19, whether these adverse effects continue after recovery and the characteristic of gut microbiota in the recovered patients are still unclear.

A better understanding of gut microbiota in recovered COVID-19 patients will be informative regarding the degree of recovery and further intervention and nursing they may need. In this study, the 16S rRNA sequencing analysis of the gut microbiota profile was performed in seven male recovered COVID-19 patients and seven healthy controls, with the goal of evaluating differences between the two groups. To the best of our knowledge, this is the first description of intestinal microbiota in recovered COVID-19 patients.

## Methods and Materials

### Participants Recruiting and Sample Collection

Participants were recruited from the Center for Reproductive Medicine, Tongji Medical College, Huazhong University of Science and Technology. Inclusion criteria for recovered COVID-19 patients included documented recovery, age from 25 to 45 years, and having detailed medical records during hospitalization and discharge certificate. The diagnosis of COVID-19 was determined by the New Coronavirus Pneumonia Prevention and Control Program (7th edition) published by the National Health Commission of China (14). Exclusion criteria included asymptomatic cases, having received antibiotics or probiotics within 2-months, gastrointestinal diseases, and severe basic diseases. Healthy controls were individuals who were enrolled at regular physical checkups in the same hospital. Ethical approval for the study was obtained by the Ethics Committee for Clinical Research of Reproductive Medicine Center, Tongji Medical College, Huazhong University of Science and Technology. Seven male cases and seven male healthy controls were recruited in this study. All participants came from Hubei province and shared a similar moderate-fat dietary habit (mainly wheat flour, rice, pork, eggs, vegetables), in which fat provided about 30% energy. Meanwhile, participants were instructed to avoid any food and drugs affecting gastrointestinal function before sampling. The collection and storage of fecal samples adhered to the relevant standards. All fecal samples were collected immediately in sterile plastic tubes and stored at −80°C until analysis. Fecal SCFAs levels were detected using the Agilent 7,890B-7,000D GC-MS/MS platform with detailed methods described in the Supplementary Methods. Blood samples were collected from participants for analyzing serum antibody and cytokines with detailed methods described in the Supplementary Methods.

### DNA Extraction and 16S rRNA Sequencing

Total bacterial genomic DNA was extracted from fecal samples using the E.Z.N.A.® soil DNA Kit (Omega Bio-tek, Norcross, GA, U.S.) according to the manufacturer's protocols. The final DNA concentrations were determined by NanoDrop 2,000 UV-vis spectrophotometer (Thermo Scientific, Wilmington, USA), Thereafter, the quality of genomic DNA was checked through 1.0% agarose gel electrophoresis. The V3–V4 region of the 16S rRNA gene was amplified using primers 338F (5'-ACT CCT ACG GGA GGC AGC AG-3') and 806R (5'-GGA CTA CHV GGG TWT CTA AT-3') on the GeneAmp 9,700 PCR System (Applied Biosystems, Foster City, CA, US). The PCR reactions were conducted according to the following program: 3 min of denaturation at 95°C, 27 cycles of 30 s at 95°C, 30 s for annealing at 55°C, and 45 s for elongation at 72°C, and a final extension at 72°C for 10 min. PCR reactions were performed in triplicate 20 μl mixture containing 4 μl of 5 × FastPfu Buffer, 2 μl of 2.5 mM dNTPs, 0.8 μl of each primer (5 μM), 0.4 μl of FastPfu Polymerase and 10 ng of template DNA. Amplification products were extracted from 2% agarose gels and purified by the DNA Gel Extraction Kit (Axygen Biosciences, Union City, CA, U.S.) and quantified by QuantiFluor ™ -ST (Promega, USA). Subsequently, the PCR amplification products were pooled in equimolar and paired-end sequenced (2 × 300) on an Illumina MiSeq platform (Illumina, San Diego, USA) at the Shanghai Majorbio Bio-pharm Technology Co., Ltd (Shanghai, China).

### Gut Microbiota Data Processing and Statistical Analysis

Raw FASTQ files were demultiplexed, quality-filtered by Trimmomatic, and merged by FLASH (15, 16) with the following criteria: (i) the reads were truncated at any site receiving an average quality score <20 over a 50 bp sliding window; (ii) primers were exactly matched allowing 2 nucleotide mismatching, and reads containing ambiguous bases were removed; (iii) sequences whose overlap longer than 10 bp were merged according to their overlap sequence. And subsequent high-quality reads were clustered into operational taxonomic units (OTUs) at 97% sequence similarity via USEARCH (Version 8.1). The taxonomy of each 16S rRNA gene sequence was classified using the Ribosomal Database Project (RDP) Classifier tool (Version 2.2) against the SILVA 119 16S rRNA database with a confidence threshold of 70%. OTUs were used for alpha diversity (Shannon, Simpson), richness (Chao) with a 97% threshold. Non-metric multidimensional analysis (NMDS) and principal coordinates analysis (PCoA) were performed based on the Bray-Curtis distance matrix calculated using OTU information from each sample. The differentially enriched microbes between two groups from phylum to genus were analyzed via the Wilcoxon rank-sum test. In addition, QIIME2- DADA2 flows were also run for reexamination, and the detailed process as well as results are available in Supplementary Materials. Statistical analyses were conducted in SPSS 26.0 (Chicago, Illinois, USA). Continuous variables were expressed as the mean ± SD and analyzed with the Student *t*-test. Categorical variables were expressed as percentages and compared by the chi-square test. *P* < 0.05 were accepted as indicating a significant difference.

## Results

### Characteristics of Recruited Recovered COVID-19 Participants

We recruited seven male recovered COVID-19 patients, and their clinical characteristics are summarized in Table 1. The mean length of hospital stay was 19.4 days, and the time between discharge and sampling from 78 to 106 days, with an average time of 90 days. Two patients had complications during the course of the disease; of note, gastrointestinal symptoms were reported in one of the patients. According to the New Coronavirus Pneumonia Prevention and Control Program (7th edition), the degree of disease severity was classified as mild, moderate, or severe. The CoV-IgG of all patients was positive and CoV-IgM was found in only one of seven patients, which means that most patients have entered the recovery phase entirely. Moreover, the pro-inflammatory cytokines IL-6 and TNF-α showed no significant differences between recovered patients and healthy people (Supplementary Figure 1). The comparison of the baseline characteristics of COVID-19 group and healthy controls is shown in Supplementary Table 1. There was no significant difference among the groups.

**Table 1:** Clinical characteristics of recruited COVID-19 patients.

**Patient no**.	**Age (yrs)**	**Disease severity**	**Disease duration (ds)**	**Time from discharge to sampling (ds)**	**Gastrointestinal symptoms**	**Comorbidity**	**CoV-IgM**	**CoV-IgG**
1	29	Severe	25	79	Diarrhea	Community-acquired pneumonia and Fatty liver	Positive	Positive
2	36	Moderate	13	83	None	Fatty liver	Negative	Positive
3	43	Mild	12	89	None	None	Negative	Positive
4	40	Severe	32	96	None	None	Negative	Positive
5	39	Mild	9	98	None	None	Negative	Positive
6	36	Moderate	24	78	None	None	Negative	Positive
7	43	Moderate	21	107	None	None	Negative	Positive

### The Gut Microbiota Compositions of Recovered COVID-19 Patients and Controls

After merging and filtering, 756,359 high-quality sequences were acquired from 14 fecal samples by 16S rDNA gene sequencing with an average sequence number of 54,025 per sample. Subsequently, 397 OTUs were clustered at a 97% similarity level. The rarefaction curves became flat, and the Shannon index also approached a clear plateau, which together suggested that a near-complete diversity has been captured from each sample (Supplementary Figures 2A,B).

**Figure 1:**
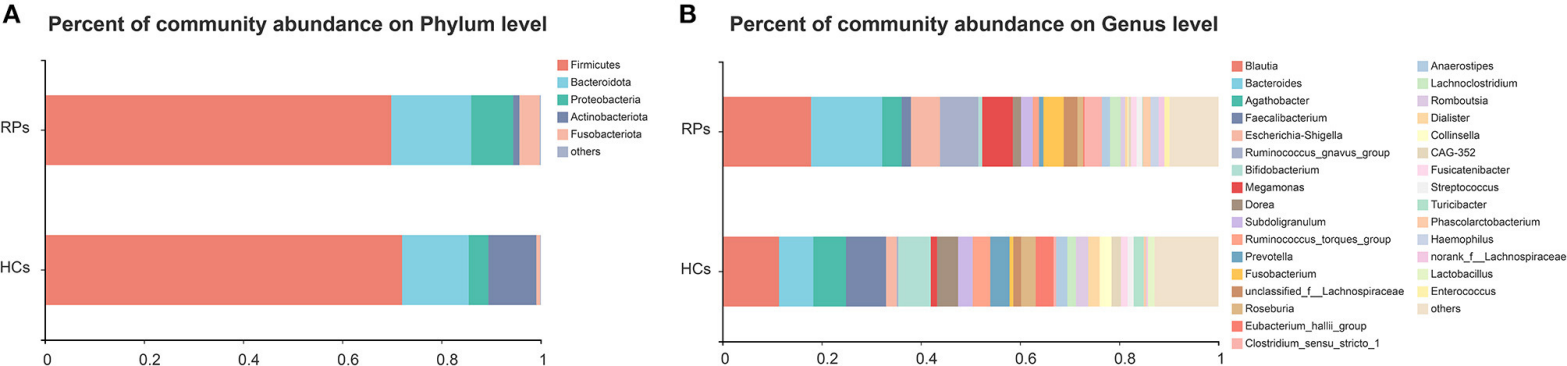
The relative abundance of gut microbiota at the Phylum **(A)** and Genus **(B)** levels in the healthy control group and the recovered COVID-19 patient group. The category “Other” covers all other phyla or genera with a low taxa abundance. RPs, recovered patients; HCs, healthy controls.

The compositions of gut microbiota of study subjects in the level of phylum and genus were shown in Figure 1A. Eight phyla were identified through bacteria microbiota analysis, and both the COVID-19 patients and healthy controls were dominated by four phyla, *Firmicutes, Bacteroidota, Proteobacteria, Actinobacteriota*, and *Fusobacteriota*. The *Firmicutes* were the predominant phylum, contributing 72.0 and 69.8% of the microbiota in healthy volunteers and COVID-19 patients, respectively. *Actinobacteriota* was the fourth most dominant phyla in healthy volunteers (9.7%), while a clear decrease in COVID-19 patients (1.3%). The second, third, and fifth most dominant phyla in healthy controls were *Bacteroidota* (13.4%), *Proteobacteria* (4.0%), and *Fusobacteriota* (0.8%); however, all of these phyla were elevated in the COVID-19 groups (*Bacteroidota* 16.1%, *Proteobacteria* 7.8%, *Fusobacteriota* 4.1%) (Figure 1A). The dominant genera of gut microbiota in both groups were *Blautia, Bacteroides, Agathobacter, Faecalibacterium*, and *Escherichia-Shigella*, with plenty of other sporadic genera (Figure 1B). The composition of the intestinal microbiota of each participant is shown in Supplementary Figure 3. Taken together, the gut microbiota of the two groups remains consistent in the overall microbiota composition but shows differences in proportions of some bacteria.

### Comparison of Microbiota Between Recovered Patients and Healthy Controls

**Figure 2:**
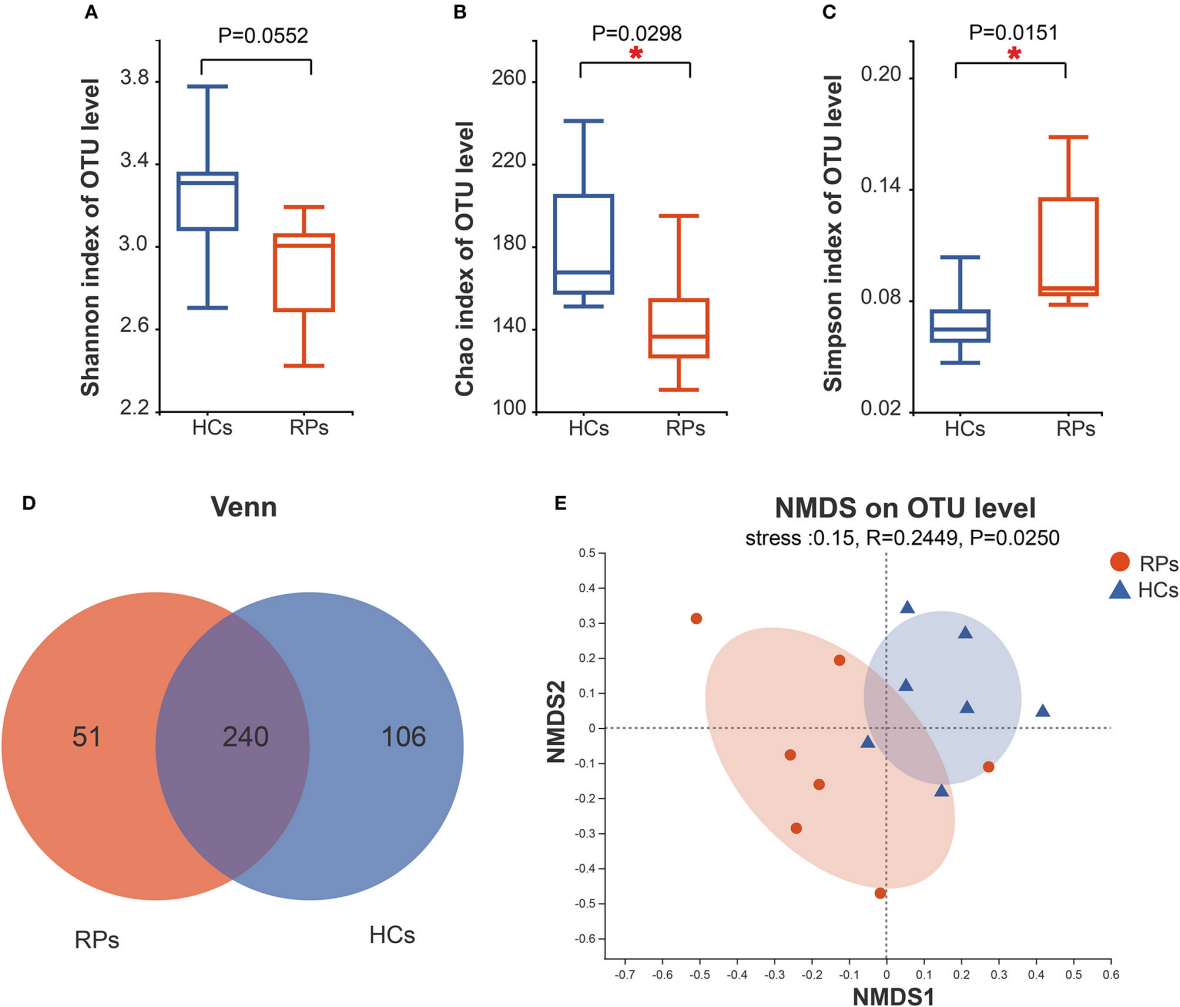
Differences in the diversity and richness of fecal microbiota between recovered COVID-19 patients and healthy controls. The Shannon **(A)**, Chao **(B)**, and Simpson index **(C)** were calculated at a 3% distance. **(D)** A Venn diagram indicates the overlapping and unique OTUs among the groups. **(E)** Non-metric multidimensional analysis (NMDS) was conducted at the OTU level to show the different distribution of recovered patients and controls.

To investigate whether an infection has a long-term effect on the gut microbiota, we evaluated the richness and diversity of recovered patients and healthy controls using the α-diversity including the Shannon index, Chao index, and Simpson index (Figures 2A–C). Recovered COVID-19 patients showed a decreasing trend in microbial diversity and richness, there was a statistically significant difference among the groups in light of richness estimator, Chao index (*p* = 0.0298), and diversity estimator Simpson index (*p* = 0.015). But there was no significant difference in another diversity estimator, the Shannon index (*p* = 0.055). Other α-diversity estimators are summarized in Supplementary Table 2. Meanwhile, the number of OTUs in the recovered patient group and health group were 291 and 346, and 240 OTUs overlapped between the two groups (Figure 2D). β-diversity was calculated through NMDS and PCoA in the level of OTUs. NMDS indicated that a clear separation between recovered patients and healthy controls (R = 0.02449, *p* = 0.0250) (Figure 2E). PCoA also produced a similar result (Supplementary Figure 4). Taken together, our results indicated a role of SARS-CoV-2 infection in affecting the fecal microbiota structure.

### Differences in Specific Microbes of Recovered COVID-19 Patients and Controls

**Figure 3:**
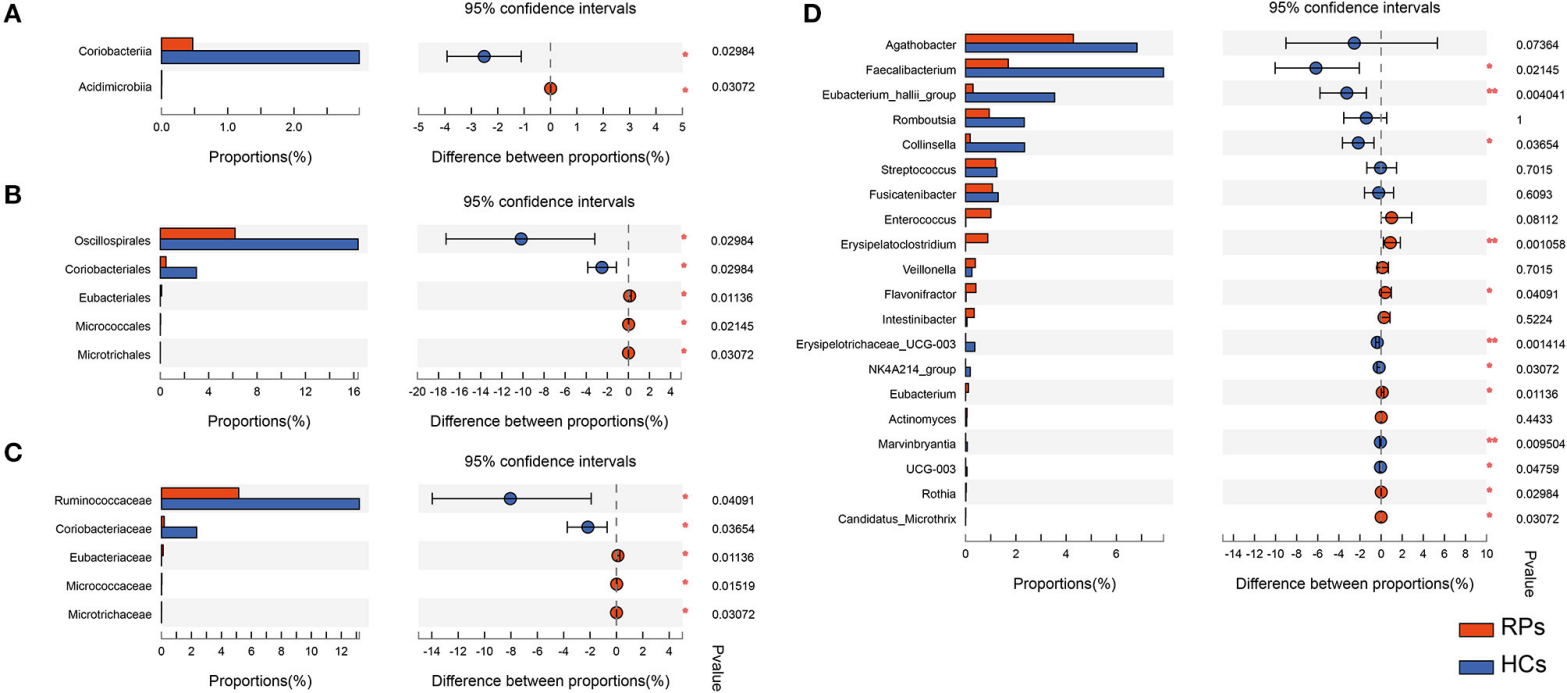
The taxonomic differences of recovered COVID-19 patients and controls in the level of Class **(A)**, Order **(B)**, Family **(C)**, and Genus **(D)**.

Many microbes differ substantially between the two groups, characterized by the decrease in beneficial bacteria and increase in opportunistic pathogens (Supplementary Table 3). There was no significant difference in the phylum level. At the class level, recovered COVID-19 patients had a significant decrease in *Coriobacteriia* (*p* = 0.030), with an increase in *Acidimicrobiia* (0.031) compared with controls, partly caused by the abundant change of order *Coriobacteriales* and *Microtrichales* (Figure 3A). Additionally, at the order level, the relative abundance of *Eubacteriales* (0.011) and *Micrococcales* (0.021) increased in the recovered patients, while *Oscillospirales* (0.030) decreased compared with the controls (Figure 3B). At the family level, the relative abundance of *Ruminococcaceae* (*p* = 0.010) and the dramatic reduction in *Coriobacteriaceae* (*p* = 0.037) in recovered patients, with an elevation in the abundance of *Eubacteriaceae* (*p* = 0.011), *Micrococcaceae* (*p* = 0.015), *Microtrichaceae* (0.031) (Figure 3C). At the Genus level, recovered COVID-19 patients showed a clear decrease in *Faecalibacterium* (0.021), *Eubacterium hallii* group (0.004), *Collinsella* (0.037), *Erysipelotrichaceae UCG-003* (0.001), *NK4A214* group (0.031), and a concomitant increase in *Flavonifractor* (0.041), *Eubacterium* (0.011), *Rothia* (0.030), *Candidatus Microthrix* (0.030), especially the *Erysipelatoclostridium* (0.001) (Figure 3D). This is partly similar to previous results on COVID-19 inpatients (7). On the one hand, a remarkable depletion was observed in commensals, particularly in the anti-inflammatory bacteria, such as *Faecalibacterium* and the *Eubacterium_hallii_*group. On the other hand, opportunistic pathogens such as *Rothia* and *Erysipelatoclostridium* showed a higher relative abundance than controls.

**Figure 4:**
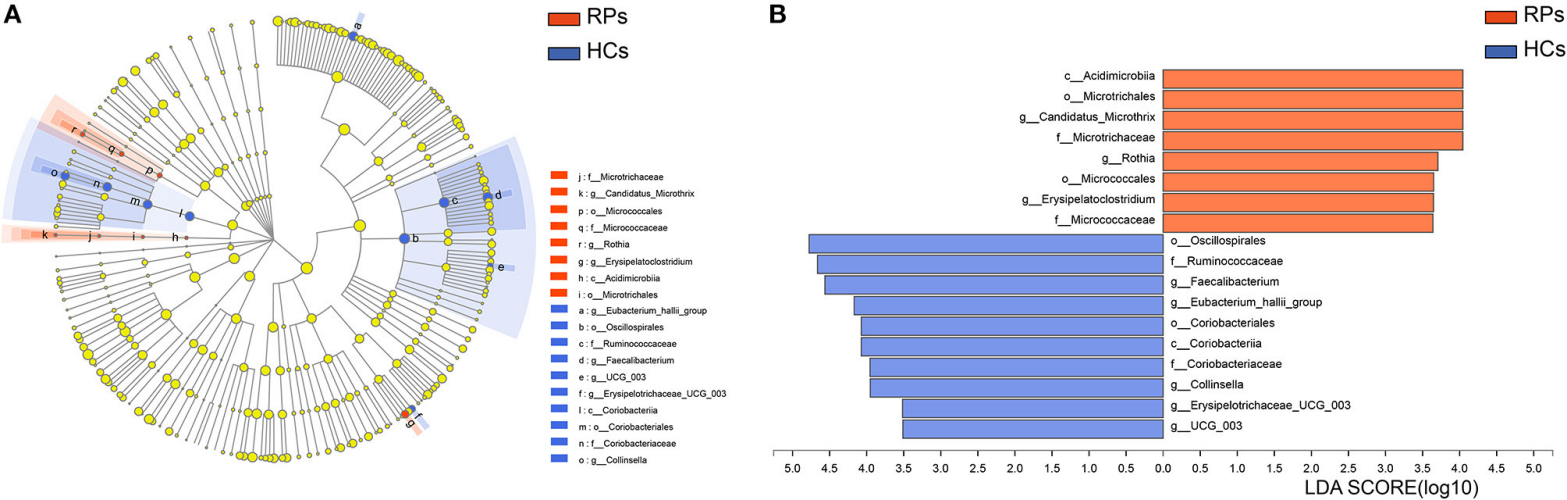
Identifying bacterial differences by the LEfSe. **(A)** Taxonomic cladogram shows the taxa that were considered statistically significant between two groups from the Phylum to the Genus level. **(B)** the LDA score of differentially enriched taxa in healthy controls and COVID-19 group (LDA threshold value >3.5).

To identify potential bacterial taxon biomarkers between recovered patient and healthy controls, linear discriminant analysis (LDA) effect size (LEfSe) was used to find species that differed significantly in abundance between the groups. A cladogram was used to show microbiota structures at the phylum and genus levels by an LDA score >3.5 (Figure 4). The gut microbiome of the COVID-19 group was mainly dominated by *Acidimicrobiia, Microtrichales, Candidatus Microthrix, Microtrichaceae, Rothia, Micrococcales, Erysipelatoclostridium*, and *Micrococcaceae*. These species may act as biomarkers to distinguish and analyze the outcomes of recovered patients on gut health.

### Levels of Fecal SCFAs in Recovered Patients and Healthy Controls

**Figure 5:**
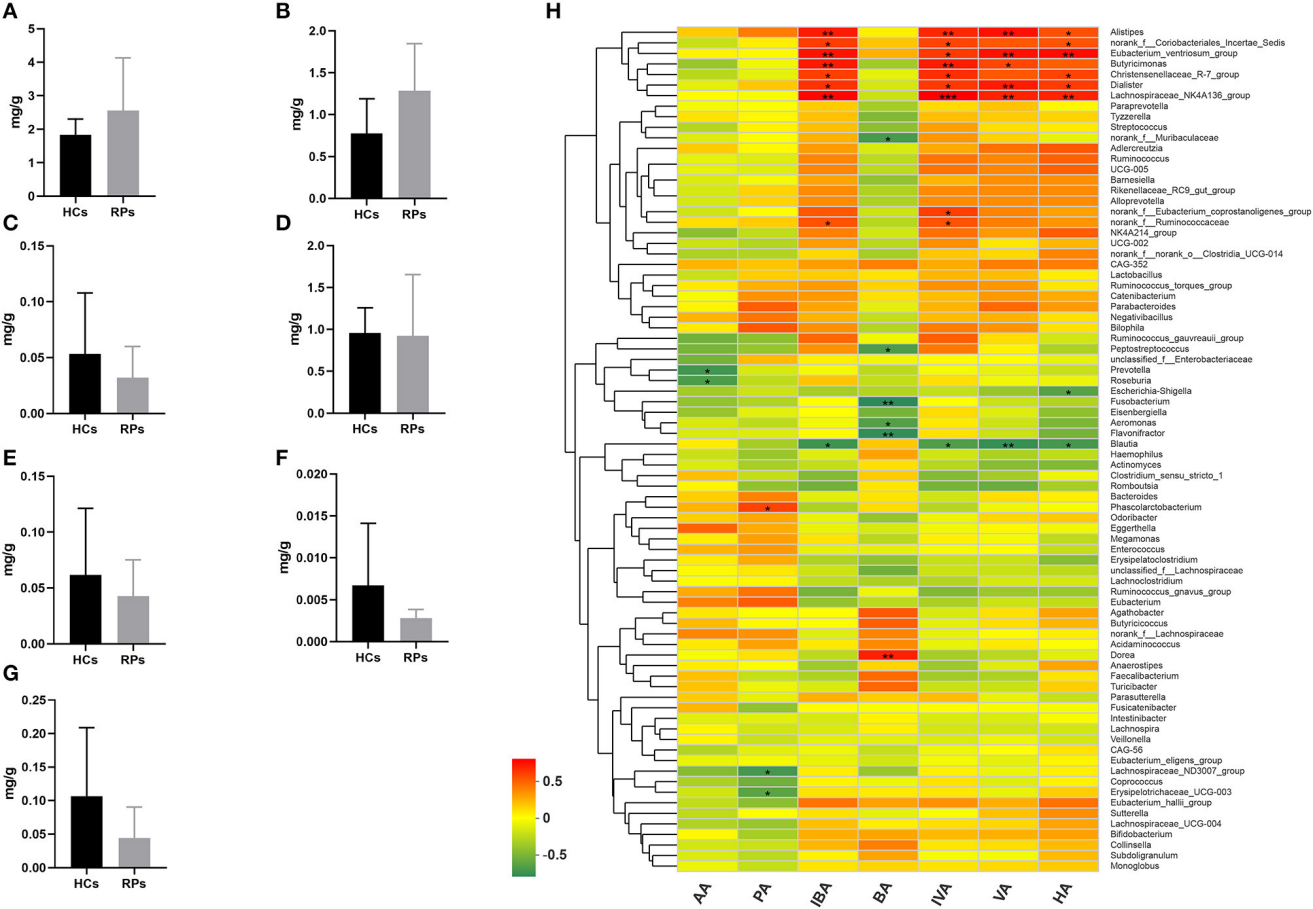
The levels of SCFAs in the fecal samples of recovered patients and healthy controls. Examination of fecal samples of the two groups on levels of **(A)** Acetic acid (AA), **(B)** Propionic acid (PA), **(C)** Isobutyric acid (IBA), **(D)** Butyric acid (BA), **(E)** Isovaleric acid (IVA), **(F)** Valeric acid (VA), and **(G)** Hexanoic acid (HA). **(H)** Correlation between relative abundance of bacteria genera and levels of seven SCFAs. *p < 0.05; **p < 0.01.

Family *Ruminococcaceae*, genus *Faecalibacterium* and *Eubacterium_hallii_group* showed a significant decrease in recovered patients and are SCFAs-producing bacteria (17–19). Decreased production of SCFAs has been found to be a consequence in mouse models of influenza A virus infection, which was capable of affecting immune responses in the lungs and modulating disease outcomes (20). Therefore, we examined the fecal concentrations of certain SCFAs, including acetic acid (AA), propionic acid (PA), isobutyric acid (IBA), butyric acid (BA), isovaleric acid (IVA), valeric acid (VA), and hexanoic acid (HA) among the two groups (Figures 5A–G). Intriguingly, there was no significant difference in the level of any SCFA. This result indicated that the SCFA levels of recovered patients were normal, though the upstream bacteria varied. We further analyzed the relationship between SCFA levels and bacterial genera using correlation heatmap based on the spearman correlation coefficient (Figure 5H). Intriguingly, there was no significant association between altered microbes and SCFAs levels. However, other SCFA-producing bacteria, such as *Alistipes, Dialister, Butyricimonas*, and *Phascolarctobacterium*, demonstrate remarkable correlation with at least one SCFA, the relative abunance of which was not decreased according to analysis mentioned above.

Taken together, the drop in abundance of several anti-inflammatory bacteria that can secrete SCFAs does not impact SCFA levels, probably because of supplementation from other SCFA-producing bacteria, and other potential impacts of reduced anti-inflammatory bacteria should be paid attention to.

## Discussion

Our study may be the first to describe the gut microbiota of people recovered from COVID-19. Compared with healthy controls, there was a clear difference in the relative abundance of gut microbiota between recovered male patients and controls. A previous report revealed that several opportunistic pathogens at the phylum level showed a clear elevation in COVID-19 inpatients, including *Streptococcus, Rothia, Veillonella, Erysipelatoclostridium*, and *Actinomyces* (7). In our study, the genera *Rothia* and *Erysipelatoclostridium* in recovered patients was also significantly more abundant than in the control group, indicating a long-term effect on gut microbiota. *Rothia* belongs to the oral-related microbes, which were correlated with Th17-induced lung inflammation and pneumonia of immunocompromised patients (21, 22). The elevation of *Rothia* in gut may suggest a possible shift of microbes from the mouth and respiratory tract to the intestines. However, increases in *Streptococcus, Actinomyces*, and *Veillonella* were observed but did not approach statistical significance in our study. These differences may suggest potential dynamic changes with the progression of COVID-19 and reflect the distinction of different stages in this disease.

We also analyzed our data by QIIME2 flow. Although there were no obvious differences in α-diversity estimators, Venn diagrams, and NMDS showed more significant separation between the two groups (Supplementary Figure 5). Additionally, the differences in relative abundance of specific microbes obtained by QIIME2 were similar to Figure 3, especially in opportunistic pathogens and SCFAs-producing bacteria (Supplementary Figures 6A–D). Besides, *Rothia, Micrococcales, Erysipelatoclostridium*, and *Micrococcaceae* were also identified using LEfSe in QIIME2 analysis (Supplementary Figures 6E,F). Taken together, results obtained from the two flows are generally consistent.

Underlying mechanisms of bacteria changes should be considered. Direct infection of virus and subsequent pathological changes including inflammation and hypoxia could be the most obvious causes of the abnormal gut microbiota (23). Besides, the use of antiviral medications and antibiotics during hospitalization also has a severe impact on the delicate balance in bacteria community. Intriguingly, ACE2, the receptor of SARS-CoV-2 also plays a role in maintaining the intestinal microbiome homeostasis, and the infection of SARS-CoV-2 may down-regulate the availability of ACE2 and induce dysfunction of the microbiota (24). The duration of these adverse effects is a key factor in evaluating the intestinal health of recovered patients. Zuo et al. demonstrated that altered gut microbiota continued after clearance of the virus when patients were discharged (8). And our study further suggests that changes in gut microbiota were not fully restored after a 3-month recovery.

Another vital question is whether these alterations in gut microbiota have a substantial impact on human health. Gut microbiota is a vital determinant of normal gut function and immunity, and potential instability may contribute to multiple diseases. SCFAs are capable of regulating the inflammatory responses by binding the G-protein-coupled receptor 43 and suppressing the activation of nuclear factor kappa-B (25, 26), which has been established as a mechanistic link between the anti-inflammation effect and the gut microbiota. Abnormality of relative abundance of some SCFAs-producing bacteria were reported in COVID-19 inpatients (7, 8). We also observed that recovered patients had a remarkable decrease in SCFA-producing bacteria, including the family *Ruminococcaceae* and genus *Faecalibacterium* and the *Eubacterium hallii* group, but the levels of SCFAs in recovered patients were normal. The supplementation from other SCFA-producing bacteria could be the vital point. Additionally, Sokol et al. (27) analyzed the dynamic changes of the gut microbiota in macaques over the entire course of SARS-CoV-2 infection. SCFA production declined during infection and returned to a normal level after recovery. Similar shifts may occur in COVID-19 patients. Notably, *Faecalibacterium* also functions in preventing inflammatory bowel disease by secreting other anti-inflammatory productions such as salicylic acid and Microbial Anti-Inflammatory Molecule (28). And the enrichment of opportunistic pathogens indicates an increased risk of chronic inflammation in the gut (18, 29). Thus, persistent monitoring of gut health in recovered patients is strongly recommended.

We acknowledge some limitations in this study. First, this is a very small sample size, and objects center on male patients, which was due to the difficulty in recruiting patients recovered from COVID-19 for just 3-months. The results provided preliminary evidence, which should be interpreted with great caution. Multicenter studies involving more cases are invited to verify our results. Second, this is a cross-sectional study in which only recovered patients were evaluated. The microbiota configuration in other stages, particularly before SARS-CoV-2 infection and during hospitalization, is helpful to reveal the thorough effect of COVID-19 on gut microbiota. Third, whether imbalanced gut microbiota causes functional impairments in recovered patients is unknown due to a lack of clinical studies. Therefore, it must be emphasized that the adverse effects that COVID-19 has induced via gut microbiota should not be overstated for now (30).

To conclude, we reported the differences of gut microbiota between recovered COVID-19 patients and controls, which propel our knowledge regarding the persistence of implications on gut microbiota mediated by COVID-19. Our findings partly support the previous studies on inpatients with COVID-19 and also indicated a possible change in gut microbiota of COVID-19 patients in different stages. The remarkable decrease of anti-inflammatory bacteria may suggest a potential risk of chronic intestinal inflammation disorders for recovered patients; thus, interventions to restore the microbiota ecology should be considered. Longstanding retrospective studies are warranted to reveal the thorough impact of COVID-19 on human health via gut microbiota and to help develop appropriate nutritional interventions for recovered patients.

## Data Availability Statement

The datasets generated for this study can be found in online repositories. The names of the repository/repositories and accession number(s) can be found at: https://www.ncbi.nlm.nih.gov/, PRJNA683685.

## Ethics Statement

The studies involving human participants were reviewed and approved by Ethics Committee for Clinical Research of Reproductive Medicine Center, Tongji Medical College, Huazhong University of Science and Technology. The patients/participants provided their written informed consent to participate in this study.

## Author Contributions

L-qZ, HL, and C-lX designed the project. YY, T-qM, ZY, S-mG, Z-mL, K-yS, and YT performed the experiments. YT and KS analyzed the data and wrote the manuscript. LZ, H-gL, and YY revised the manuscript. All authors contributed to the article and approved the submitted version.

## Conflict of Interest

The authors declare that the research was conducted in the absence of any commercial or financial relationships that could be construed as a potential conflict of interest.
