# Supplementary material for: Gut Microbiota May Not Be Fully Restored in Recovered COVID-19 Patients After 3-Month Recovery
Source: Front Nutr. 2021 May 13;8:638825. doi: 10.3389/fnut.2021.638825 (PMC8155354; doi:10.3389/fnut.2021.638825)
Supplement: Supplementary file 1 [file Data_Sheet_1.docx]

Supplementary Material

## Supplementary Methods

**1.**Determination of levels of short chain fatty acids (SCFAs)

1.1 Chemicals and reagents

Methyl tert-butyl ether (MTBE) were purchased from CNW (CNW Technologies, Germany). MilliQ water (Millipore, Bradford, USA) was used in all experiments. All of the standards were purchased from CNW (Beijing) or Aladdin (Shanghai). The stock solutions of standards were prepared at the concentration of 1 mg/mL in MTBE. All stock solutions were stored at -20 °C. The stock solutions were diluted with MTBE to working solutions before analysis.

1.2 Sample preparation and extraction

20 mg of fecal samples were accurately weighed and placed in a 2 ml EP tube. 1 mL of phosphoric acid (0.5% v/v) solution and a small steel ballwere added to the EP tube. The mixture wasgrinded for 10s,three times, then vortexed for 10 minutes and ultrasonicated for 5 minutes. 0.1 mL of supernatant was added to 1.5 mL centrifugal tube after the mixture was centrifuged at 12000 rpm for 10 minutes at the temperature of 4°C. 0.5 mL MTBE (containing internal standard) solution was added to the centrifugal tube. The mixture was vortexed for 3 minutes and ultrasonicated for 5 minutes. After that, the mixture was centrifugedat 12000 rpm for 10 minutes at the temperature of 4°C. The supernatant was collected and used for GC-MS/MS analysis.

1.3GC-MS analysis

Agilent 7890B gas chromatograph coupled to a 7000D mass spectrometer with a DB-FFAP column (30 m length × 0.25 mm i.d. × 0.25 μm film thickness, J&W Scientific, USA) was employed for GC-MS/MS analysis of SCFAs. Helium was used as carrier gas, at a flow rate of 1.2 mL/min. Injection was madein the split mode and the injection volume was 2 μL. The oven temperature was held at 90°Cfor 1 min, raised to 100°C at a rate of 25°C/min, raised to 150°C at a rate of 20°C/min,hold on 0.6 min, raised to 200°C at a rate of 25°C/min, hold on 0.5min, after running for 3min. All samples were analyzed in multiple reaction monitoring mode. The injector inlet and transfer line temperature were 200 °C and 230 °C, respectively.

**2.**Determination of serum antibodies and cytokines

Blood samples were collected byvenepuncture, and serum was centrifuged from samplesimmediately thesamples and stored at -80°Cimmediately for further analysis.Antibodies and proinflammatory factorsconcentrations were determined with ELISA kits (Cusabio, Wuhan, China) following the manufacturer's protocol (IgG, CSB-EL33241HU; IgM,CSB-EL33242HU ;IL-6, CSB-E04638h; tumor necrosis factorα, CSB-E04740h).

**3.**Amplicon sequence processing and analysis of QIIME2

After demultiplexing, the resulting sequences were merged with FLASH (v1.2.11)[1] and quality filtered with fastp (0.19.6)[2]. Then the high-quality sequences were de-noised using DADA2[3-4] plugin in the Qiime2[5] (version 2020.2) pipeline with recommended parameters, which obtains single nucleotide resolution based on error profiles within samples. DADA2denoised sequences are usually called amplicon sequence variants (ASVs). To minimize the effects of sequencing depth on alpha and beta diversity measure, the number of sequences from each sample was rarefied to 4000, which still yielded an average Good’s coverage of 97.90%. Taxonomic assignment of ASVs was performed using the Naive bayes consensus taxonomy classifier implemented in QIIME2 and the SILVA 16S rRNA database (v138). Analyses of the 16S rRNA microbiome sequencing data was performed using the free online platform of Majorbio Cloud Platform (www.majorbio.com).

**Reference**

[1] Magoc Tanja, Salzberg S L. FLASH: fast length adjustment of short reads to improve genome assemblies[J]. Bioinformatics(21):21.

[2] Chen Shifu, Zhou Yanqing, Chen Yaru, et al. fastp: an ultra-fast all-in-one FASTQ preprocessor[J]. Bioinformatics, 2018, 34(17):i884-i890.

[3] Callahan B J, Mcmurdie P J, Rosen M J, et al. DADA2: High-resolution sample inference from Illumina amplicon data[J]. Nature Methods, 2016, 13(7): 581-583.

[4] Amir A, McDonald D, Navas-Molina JA, et al. Deblur rapidly resolves single-nucleotide community sequence patterns. mSystems. 2017, 2:e00191–00116.

[5] Bolyen E, Rideout JR, Dillon MR, et al. Reproducible, interactive, scalable and extensible microbiome data science using QIIME 2[J]. 2019, Nature Biotechnology 37: 852-857.

# SupplementaryTables

**Supplementary Table 1.** Characteristics of the Study Subjects.

|  | Recovered patients | Healthy controls | P-value |
| --- | --- | --- | --- |
| Age(yrs) | 38.0 (4.9) | 32.8 (5.7) | >0.05 |
| Male sex | 7(100.0%) | 7(100.0%) | >0.05 |
| BMI | 26.0(3.5) | 23.3(1.7) | >0.05 |
| Gastrointestinal symptoms | 1(14.3%) | 0(0%) | >0.05 |
| Comorbidity | 2(28.6%) | 0(%) | >0.05 |

**Supplementary Table 2.** Summary of α diversity estimators.

| Estimators | RPs-Mean | RPs-Sd | HCs-Mean | HCs-Sd | P-value |
| --- | --- | --- | --- | --- | --- |
| Shannon | 2.8732 | 0.27762 | 3.2375 | 0.34304 | 0.05528 |
| Chao | 143.48 | 27.578 | 183.54 | 34.721 | 0.02984 |
| Simpson | 0.1095 | 0.040032 | 0.068678 | 0.018663 | 0.01519 |
| Coverage | 0.99952 | 0.00016389 | 0.99948 | 5.91E-05 | 0.2013 |
| Ace | 145.2 | 28.83 | 186.3 | 29.962 | 0.05528 |

**Supplementary Table 3.**Variance of the microbes present in recovered patients and healthy controls.

| Species name | RPs-Mean(%) | RPs-Sd(%) | HCs-Mean(%) | HCs-Sd(%) | P-value |
| --- | --- | --- | --- | --- | --- |
| Class | | | | | |
| c__Coriobacteriia | 0.4719 | 0.4678 | 2.985 | 1.947 | 0.02984 |
| c__Acidimicrobiia | 0.00495 | 0.006899 | 0 | 0 | 0.03072 |
| Order | | | | | |
| o__Oscillospirales | 6.179 | 4.922 | 16.35 | 8.936 | 0.02984 |
| o__Coriobacteriales | 0.4719 | 0.4678 | 2.985 | 1.947 | 0.02984 |
| o__Eubacteriales | 0.1127 | 0.1898 | 0 | 0 | 0.01136 |
| o__Micrococcales | 0.02599 | 0.01825 | 0.007205 | 0.005991 | 0.02145 |
| o__Microtrichales | 0.00495 | 0.006899 | 0 | 0 | 0.03072 |
| Family | | | | | |
| f__Ruminococcaceae | 5.155 | 5.193 | 13.2 | 7.218 | 0.04091 |
| f__Coriobacteriaceae | 0.1811 | 0.3377 | 2.348 | 2.169 | 0.03654 |
| f__Eubacteriaceae | 0.1127 | 0.1898 | 0 | 0 | 0.01136 |
| f__Micrococcaceae | 0.02439 | 0.01782 | 0.006631 | 0.006044 | 0.01519 |
| f__Microtrichaceae | 0.00495 | 0.006899 | 0 | 0 | 0.03072 |
| Genus | | | | | |
| g__Agathobacter | 4.287 | 8.597 | 6.822 | 6.062 | 0.07364 |
| g__Faecalibacterium | 1.698 | 2.513 | 7.875 | 4.984 | 0.02145 |
| g__Eubacterium_hallii_group | 0.2939 | 0.5264 | 3.543 | 3.254 | 0.004041 |
| g__Romboutsia | 0.9358 | 1.062 | 2.333 | 2.813 | 1 |
| g__Collinsella | 0.1811 | 0.3377 | 2.344 | 2.165 | 0.03654 |
| g__Streptococcus | 1.188 | 1.631 | 1.244 | 1.403 | 0.7015 |
| g__Fusicatenibacter | 1.065 | 1.561 | 1.293 | 1.297 | 0.6093 |
| g__Enterococcus | 1.006 | 2.466 | 0.008105 | 0.01242 | 0.08112 |
| g__Erysipelatoclostridium | 0.8806 | 1.291 | 0 | 0 | 0.001058 |
| g__Veillonella | 0.3817 | 0.5618 | 0.2485 | 0.4436 | 0.7015 |
| g__Flavonifractor | 0.4062 | 0.7661 | 0.01813 | 0.02156 | 0.04091 |
| g__Intestinibacter | 0.3387 | 0.7009 | 0.05688 | 0.05974 | 0.5224 |
| g__Erysipelotrichaceae_UCG-003 | 0.000999 | 0.002643 | 0.3636 | 0.2335 | 0.001414 |
| g__NK4A214_group | 0 | 0 | 0.182 | 0.3052 | 0.03072 |
| g__Eubacterium | 0.1127 | 0.1898 | 0 | 0 | 0.01136 |
| g__Actinomyces | 0.05513 | 0.03039 | 0.04032 | 0.01034 | 0.4433 |
| g__Marvinbryantia | 0.001073 | 0.00204 | 0.06346 | 0.09271 | 0.009504 |
| g__UCG-003 | 0.000686 | 0.001815 | 0.05449 | 0.1174 | 0.04759 |
| g__Rothia | 0.02336 | 0.01898 | 0.006631 | 0.006044 | 0.02984 |
| g__Candidatus_Microthrix | 0.00495 | 0.006899 | 0 | 0 | 0.03072 |

RPs, recovered patients; HCs, healthy controls; Sd, Standard deviation.

**Supplementary Figures**


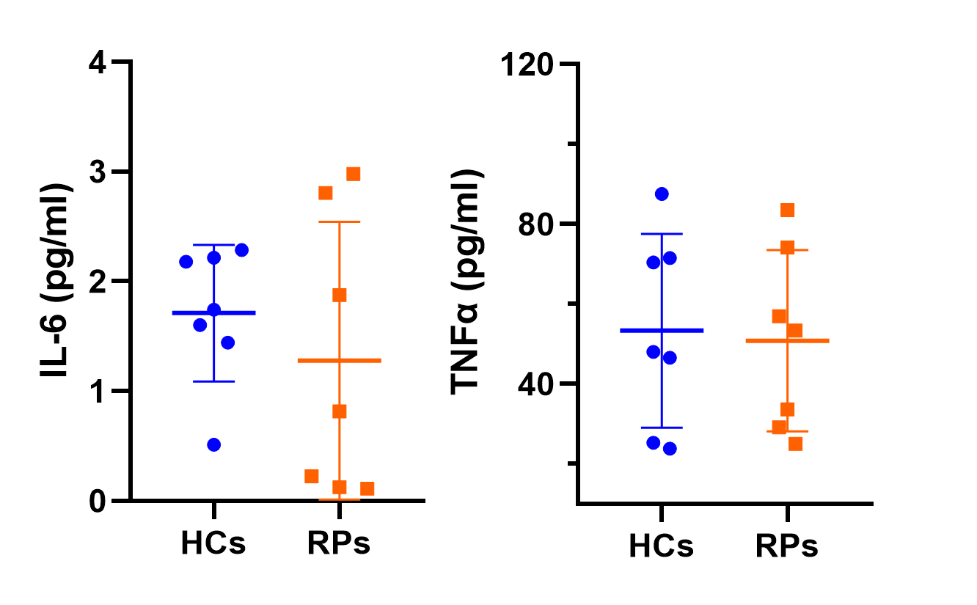


**Supplementary Figure 1**. Levels of IL-6 and TNFα in serum from healthy people and recovered patients. HCs are marked by blue, and RPs are marked by orange.


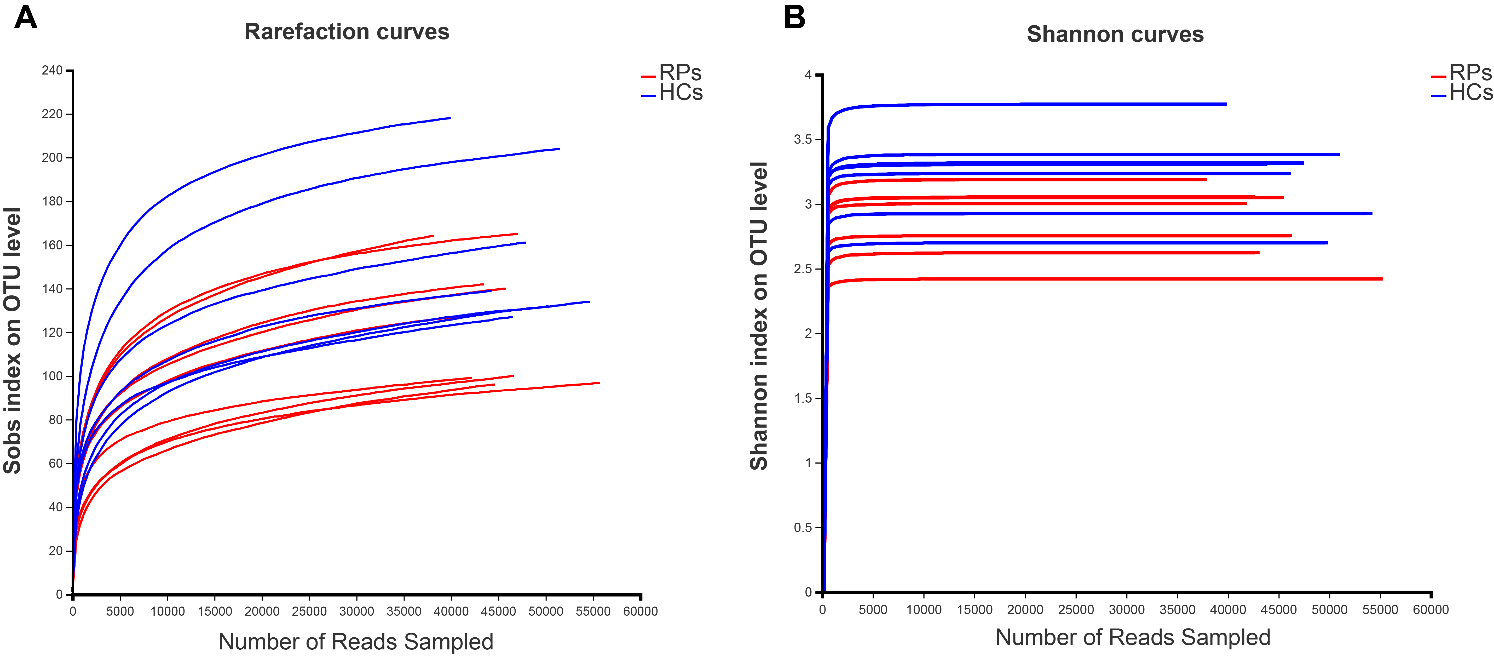


**Supplementary Figure 2.** Rarefaction curve (A) and Shannon curve (B) of each sample.


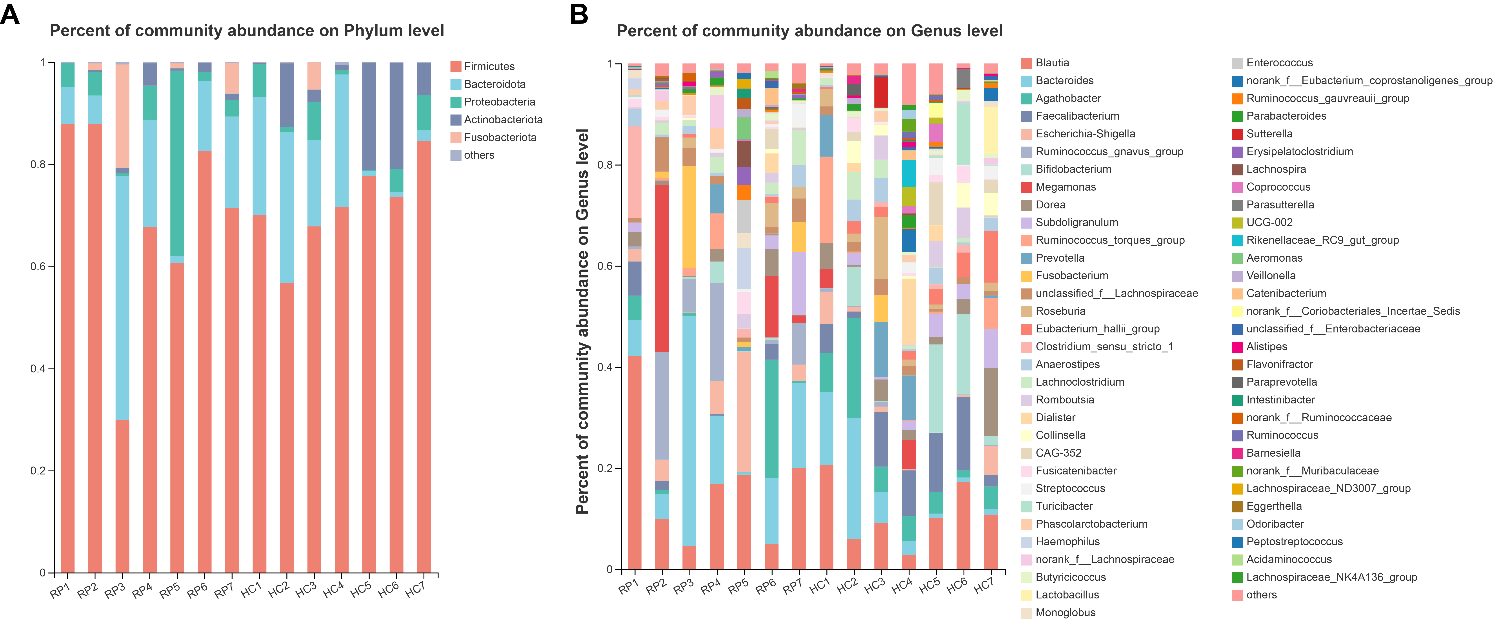


**Supplementary Figure 3**. Gut microbiota configuration at Phylum (A) and Genus (B) level in every participant. The category ‘Other’ covers all other phyla or genera with a low taxa abundance.


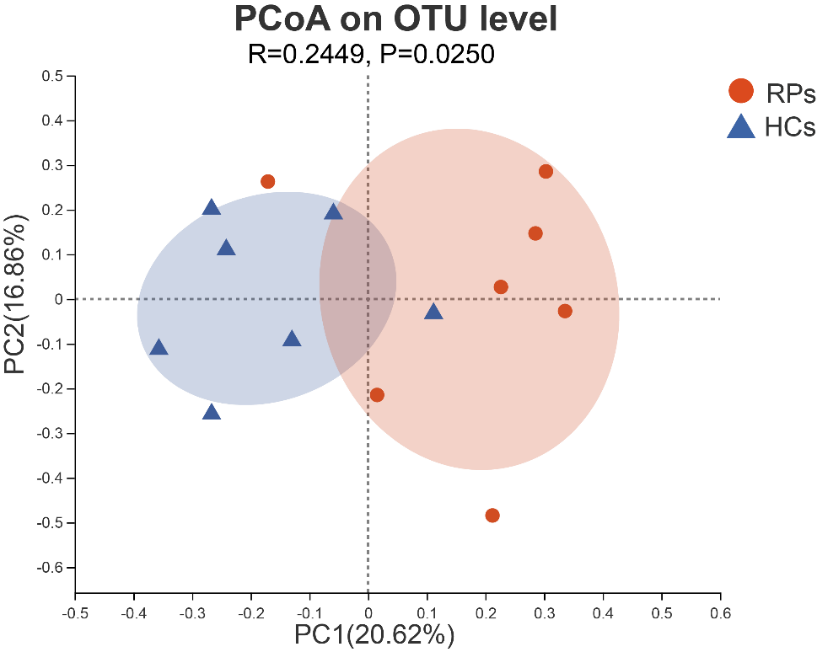


**Supplementary Figure 4.**Principal coordinates analysis (PCoA) was conducted at the OTU level to present the different distribution of healthy people and recovered patients.


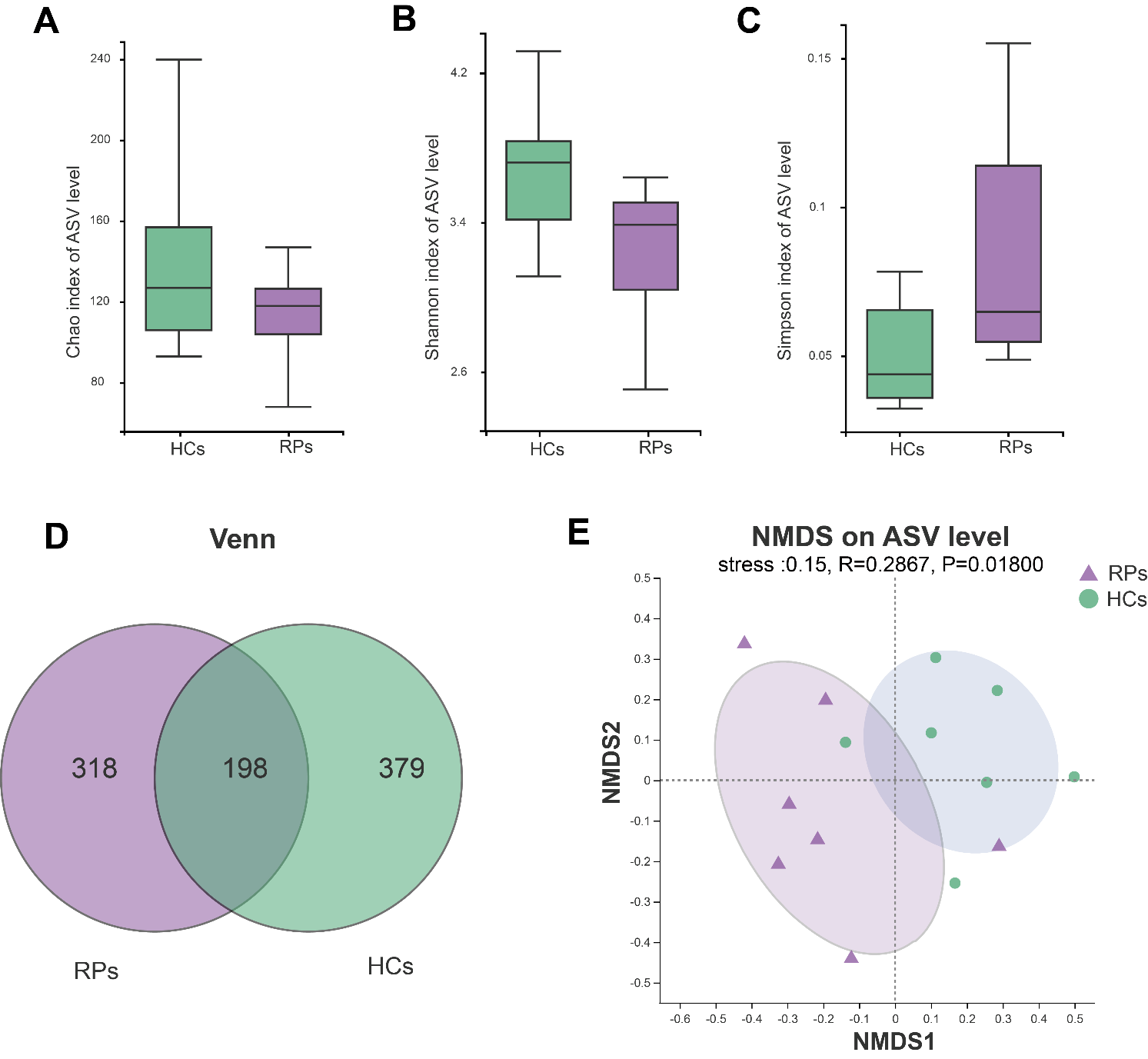


**Supplementary** **Figure 5**. α diversity estimators(A-C)and β diversity estimators (D-E)analyzed by QIIME2.

­­­­­­­­­
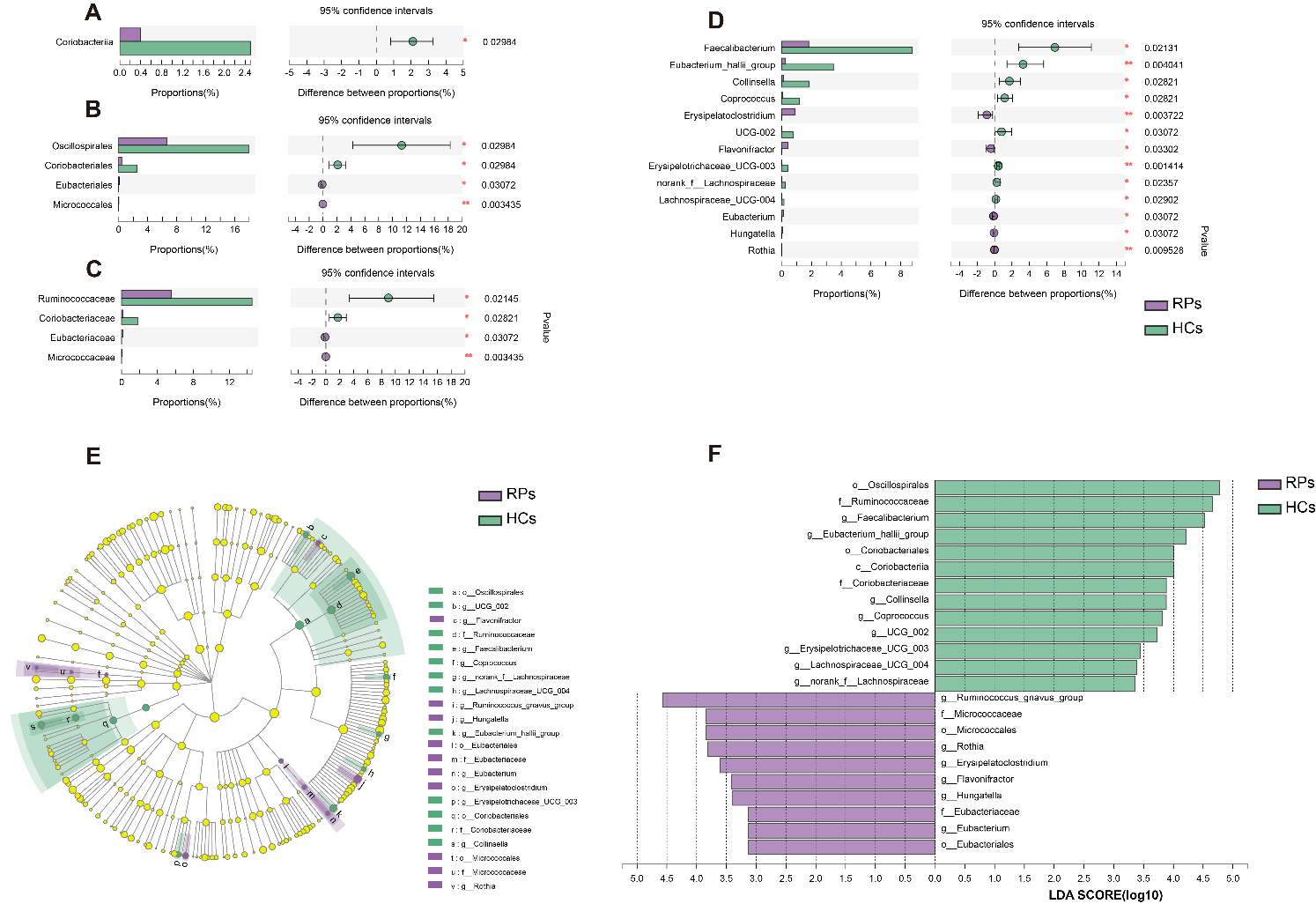


**Supplementary Figure 6**. The taxonomic differences among two groups in the level of Class (A), Order (B), Family (C), and Genus(D), and the LEfSe(E-F) analyzed by QIIME2.
